# Validation and Quality Assessment of the Kilimanjaro Cancer Registry

**DOI:** 10.1200/JGO.2015.002873

**Published:** 2016-04-27

**Authors:** Leah L. Zullig, Kristin Schroeder, Pilli Nyindo, Theresia Namwai, Elvis Silayo, Angelah Msomba, Michael Oresto Munishi, Francis Karia, Charles Muiruri, John Bartlett, Venance Maro, S. Yousuf Zafar

**Affiliations:** **Leah L. Zullig**, Durham Veterans Affairs Center for Research in Primary Care; **Leah L. Zullig**, **Kristin Schroeder**, **Charles Muiruri**, **John Bartlett**, and **S. Yousuf Zafar**, Duke University, Durham, NC; **Kristin Schroeder**, Bugando Medical Centre, Mwanza; and **Pilli Nyindo**, **Theresia Namwai**, **Elvis Silayo**, **Angelah Msomba**, **Michael Oresto Munishi**, **Francis Karia**, **Charles Muiruri**, **John Bartlett**, and **Venance Maro**, Kilimanjaro Christian Medical Centre, Moshi, Tanzania.

## Abstract

**Purpose:**

Global cancer burden has increasingly shifted to low- and middle-income countries and is particularly pronounced in Africa. There remains a lack of comprehensive cancer information as a result of limited cancer registry development. In Moshi, Tanzania, a regional cancer registry exists at Kilimanjaro Christian Medical Center. Data quality is unknown. Our objective was to evaluate the completeness and quality of the Kilimanjaro Cancer Registry (KCR).

**Methods:**

In October 2015, we conducted a retrospective review of KCR by validating the internal consistency of registry records with medical and pathology records. We randomly sampled approximately 100 total registry cases. Four reviewers not associated with the KCR manually collected data elements from medical records and compared them with KCR data.

**Results:**

All 100 reviewed registry cases had complete cancer site and morphology included in the registry. Six had a recorded stage. For the majority (n = 92), the basis of diagnosis was pathology. Pathology reports were found in the medical record for 40% of patients; for the remainder, these were stored separately in the pathology department. Of sampled registry cases, the KCR and medical records were 98% and 94% concordant for primary cancer site and morphology, respectively. For 28%, recorded diagnosis dates were within 14 days of what was found in the medical record, and for 32%, they were within 30 days.

**Conclusion:**

The KCR has a high level of concordance for classification and coding when data are retrieved for validation. This parameter is one of the most important for measuring data quality in a regional cancer registry.

## INTRODUCTION

Cancer presents a significant burden around the globe and is largely indiscriminant of economic status. Worldwide estimates suggest that in 2012, there were approximately 14.1 million new cancer diagnoses and 8.2 million cancer-related deaths.^1^ In recent years, the cancer burden has shifted and is increasingly shouldered by low- and middle-income countries (LMICs). Cancer in LMICs now accounts for about 57% of cancer cases and 65% of cancer-related deaths worldwide.^1^ By 2030, this proportion is estimated to reach 70%.^2^ The increasing burden of cancer is particularly pronounced in Africa,^3^ with about three quarters of new cancer diagnoses and deaths occurring in sub-Saharan African countries,^4^ which creates a unique set of challenges.

Comprehensive and reliable information about cancer incidence in Africa is needed. Cancer registries are a crucial source of this information, yet major gaps exist in cancer registration coverage. Merely 1% of the African population is covered under a cancer registry,^5^ and the completeness and quality of existing African cancer registry data largely have been unexplored.^6^ Moreover, simply having cancer registration is insufficient. For cancer registry data to have utility for cancer control planning and policy decisions, the quality and accuracy of the data must be assessed.

To develop a comprehensive understanding of the cancer burden in sub-Saharan Africa, population-based cancer registries are the gold standard.^4^ The development of a population-based cancer registry requires multiple steps: define the geographic area and population to be covered, have official population estimates for that group, access individual medical records and other source documents (eg, death certificates, chemotherapy records), and build continuous partnerships with local sources of information to continue data flow.^7^ Although this goal is important and attainable, hospital-based and regional cancer registries that produce quality data may be an important and intermediate step.^8^ This is particularly the case when hospital-based and regional cancer registries develop standard definitions that enable data linkages and aggregation of information across multiple registries.^9^

Our objective was to evaluate the quality of an existing regional cancer registry based at Kilimanjaro Christian Medical Center in Moshi, Tanzania.^10,11^ Specifically, we evaluated the validity of codes and classifications. The Kilimanjaro Cancer Registry (KCR) began data collection in August 2013, with the first year of complete data collection in 2014. Since, the KCR has collected information on > 3,000 unique patients with cancer. The KCR covers a population of approximately 1.64 million people who live in the Kilimanjaro region.^12^ Although the KCR makes an important contribution to understanding the cancer burden in the Kilimanjaro region of Tanzania, the quality of the data are unknown.

Two cancer registrars (both trained nurses) collect KCR data. On an ongoing basis, cancer registry data elements are abstracted from medical record charts and pathology records onto paper data collection forms. All medical charts and records are paper based and written in English. This abstracted information is later entered into CanReg5, an open-source tool developed and provided by the International Association of Cancer Registries (IACR).^13^ Patient data generally are entered into the KCR based on pathology results, and the Kilimanjaro Christian Medical Center provides centralized pathology review for the region; hence, the KCR is a regional registry. Although these data have been consistently collected for > 1 year, they have not been broadly used for clinical or policymaking purposes because their quality are unknown.

## METHODS

**Fig 1:**
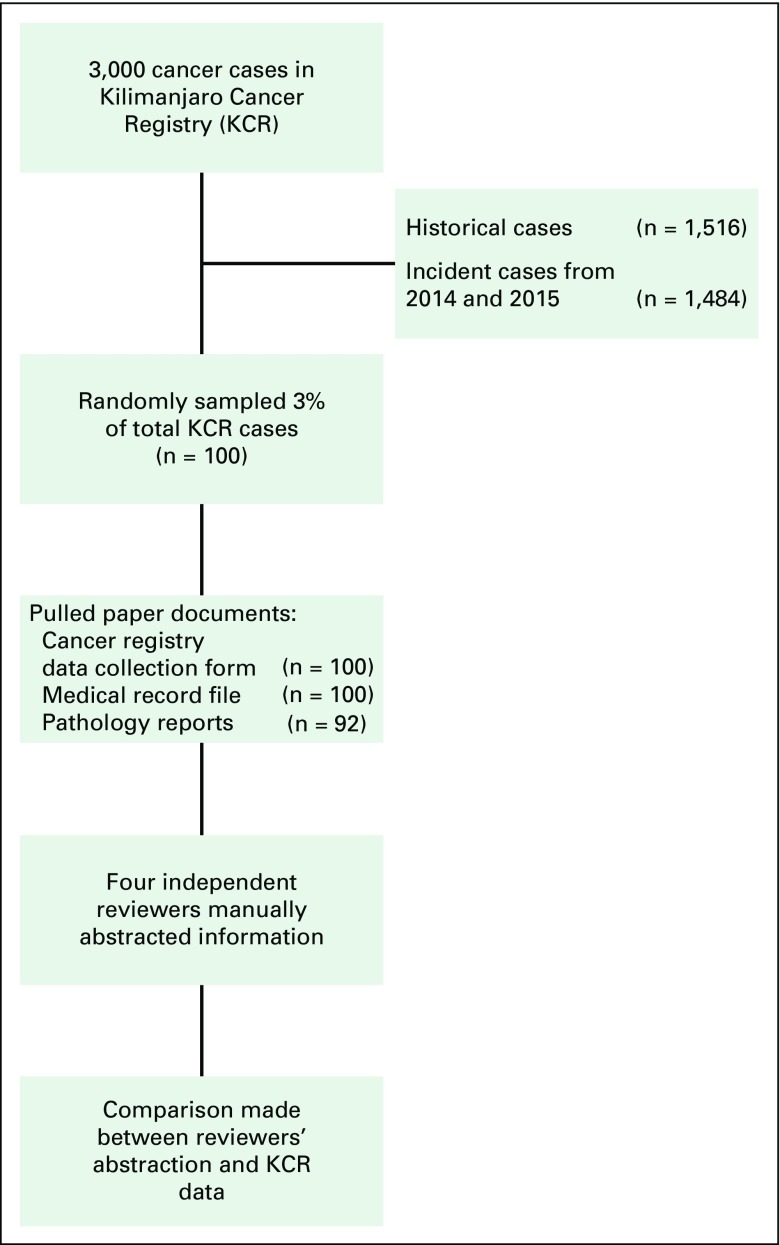
Validation process.

In October 2015, we conducted a retrospective chart review at Kilimanjaro Christian Medical Center. By using the KCR paper data collection forms, we randomly sampled 100 registry cases that were abstracted during calendar years (ie, incidence cases) 2014 to 2015 (Fig 1), which equated to approximately 3% of total cases in the KCR. To validate the registry data, we reviewed and compared the initial KCR data collection form and associated medical record charts. Although pathology is the most common data source, autopsy information, diagnostic imaging, clinical notes, and cytology data sources may also have been used as the basis of diagnosis in the KCR. Because reports from the departments of ophthalmology and pathology were not integrated with the primary medical record chart, we separately extracted charts and collected information from those departments. Therefore, the collection of complete data for a specific cancer registry case might have required data collection from several physical locations in the medical center.

Four independent reviewers not affiliated with the KCR (L.L.Z., E.S., A.M., S.Y.Z.) manually reviewed and compared the KCR data collection form with patient paper-based medical record charts and associated documents (eg, pathology reports). The reviewers have expertise in the field of medicine, specifically medical oncology and cancer health services research. By using electronic spreadsheets, the reviewers abstracted the following patient demographic characteristics: name, tribe, and geographic district of residence. Patient age or sex were not compared. Reviewers also abstracted the following information specific to patients’ diseases: cancer site, stage at diagnosis, diagnosis date, morphology, laboratory accession number (used for tracking purposes), vital status, and date of death. Where information was ambiguous, the reviewers reached consensus before recording it to ensure uniformity.

We adapted SEER coding rules to adjust for missing date information.^14^ When the day of the month was missing, we set the day to the middle of the month (eg, February 15). When the month of the year was missing, we set the month to the middle of the year (eg, June). This method made comparisons across KCR and medical record charts possible when only partial date data were available.

Data quality was evaluated by assessing completeness (defined as percentage of variables completed within a registry case) and accuracy (defined as internal consistency within a registry record and alignment with the patient’s medical record). During this review process, particular emphasis was placed on variables critical for clinical care decision making and proper cancer epidemiology reporting, such as primary cancer site; morphology; and, when possible, stage of disease. Data collection, management, and analysis were conducted in Microsoft Excel 2011 (Microsoft Corporation, Redmond, WA) and Stata 11 (StataCorp, College Station, TX) software.

## RESULTS

### KCR Completeness

The KCR contains information on > 3,000 unique patients with cancer from 32 tribal groups that live in eight geographic districts in the Kilimanjaro region of Tanzania (Table 1). We successfully located primary medical record charts for all sampled patients (n = 100). Pathology reports were inconsistently stored with the primary medical record. We identified pathology reports stored with the medical record for 40% (n = 40) of patients and obtained pathology reports from the pathology department for 59% (n = 59). Pathology reports were appropriately unavailable for one patient in whom the diagnosis was made clinically without biopsy, and a pathology report was missing.

**Table 1:**
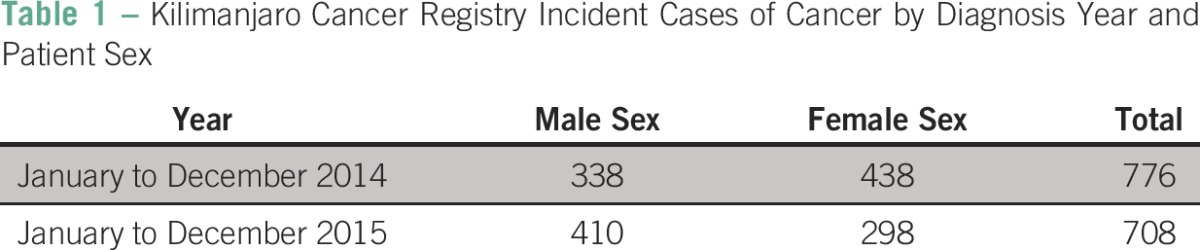
Kilimanjaro Cancer Registry Incident Cases of Cancer by Diagnosis Year and Patient Sex

The majority (92% [n = 92]) of patients had a histologic basis for cancer diagnosis. Cancer was also diagnosed based on the histology of metastases (n = 2), cytology (n = 2), and clinical information and/or imaging studies (n = 3). The basis of diagnosis was missing for one patient. Seventeen patients had partial diagnosis dates whereby the diagnosis date had missing or estimated information (eg, month and year available, but day of the month was missing or estimated). However, the date of diagnosis was only missing for one KCR patient. For all sampled KCR records, the cancer site was complete, and for those with pathology information available, morphology was also complete.

### KCR Accuracy

Among the sampled KCR records, the most commonly reported cancer sites were eye and prostate (n = 16 for each), cervix (n = 14), and breast (n = 7). These cancers are not necessarily the most common in the KCR but were the most common among the sampled patients. By comparing the medical record chart and KCR collection form, cancer site was concordant for 98% (n = 98) of patients. Similarly, when a date of death was available, it was concordant for 97% (n = 97). Morphology was concordant for 94% (n = 94) of patients. The greatest potential source of discordance between the medical record chart and KCR was date of diagnosis. However, for 28% (n = 28) of patients, recorded diagnosis dates were within 14 days of what was found in the medical record, and for 32%, these were within 30 days.

No duplicate records were identified during the review. The KCR has a three-step system for identifying and reconciling duplicate records. The first occurs as a search in the KCR for records with the same medical record number at the point of data entry. The second step involves communication with the medical records department about potential patient cases with multiple associated medical record numbers. The third step involves the production of a report to identify duplicated records in the KCR software.

## DISCUSSION

We found that the KCR had pathology reports available for most registered patients; however, the location of those reports was variable (ie, within medical records or pathology departments). Accordingly, the majority of registered patients had been given a histologically confirmed diagnosis. Cancer site and morphology generally were consistent between the KCR and medical records, but there was considerable discordance with regard to the date of diagnosis.

In developed countries, cancer registries often can rely on an existing clinical and health information technology infrastructure to support their efforts. In LMICs like Tanzania, however, this infrastructure may not be in place.^15^ An example with particular relevance to cancer registration is the delivery of pathology services. Pathology is a critical component of cancer care because it is essential for initial diagnoses and subsequent clinical decision making. Pathology services often are inaccessible in developing countries, however.^16^ In Tanzania, only 22 pathologists in the public sector serve a population of 48 million.^16^ At Kilimanjaro Christian Medical Center, visiting pathologists who come for short stays from other developed countries staff the pathology department. The dearth of pathology services is one example of the limited health care infrastructure that affects cancer registration capabilities. When health care facilities and other resources are limited, even when cancer registration occurs, the data quality may be uncertain.^7^ The lack of cancer data may delay governmental funding or development of cancer control plans^15^ and thus perpetuate the problem.

To combat this problem and to produce reliable and comparable cancer incidence information, all cancer registries (population, regional, or hospital based) must adopt and adhere to standardized coding rules and procedures, including quality standards.^7,17^ The International Agency for Research on Cancer (IACR) has developed recommendations on data elements to be collected and their standard definitions.^7^ Moreover, the IACR has established that all cancer registries provide objective measures for four dimensions of quality: comparability, validity, timeliness, and completeness.^18^ We assert that registries should strive to meet these recommendations. Additionally, we affirm that data assurance checks are necessary at multiple stages throughout the process to ensure high quality.

Another organization that provides guidance to cancer registries, the North American Association of Central Cancer Registrars, recommends that central cancer registries conduct many different types of audits at varying points in time.^19^ These audits include case-finding audits, in which central registries perform an independent review of case-finding sources for reporting facilities approximately every 3 years; reabstracting audits, when tumor information is independently abstracted from the source patient record, coded, and compared with abstracted material; and recoding audits, in which codes are assigned to abstracted text information without consulting the source records.^19^ The audit that we conducted was a simplified internal reabstracting audit. Our goal is to perform an annual audit of the KCR, which could be used to compare the quality of the KCR with other African cancer registries.

Several African cancer registries have performed quality assurance audits, notably Nigeria, The Gambia, and Uganda.^9,20,21^ Approximately 12 of Nigeria’s 24 cancer registries participated in an evaluation of completeness, comparability, and diagnostic validity that largely was focused on children’s age-specific incidence.^9^ The study found that the registration processes of the Nigeria cancer registries generally were consistent across facilities and concordant with international standards.^9^ The evaluators found high rates of diagnostic validity but also uncovered evidence of incompleteness.^9^ The Gambia is one of few nationwide, population-based cancer registries in Africa.^20^ Its cancer registry quality assessment determined that records were morphologically verified for approximately 18% and 33% of cancers among men and women, respectively, and overall completeness was estimated at approximately 50%.^20^ Among patients registered in Kampala, Uganda, completeness was much higher at nearly 90%.^21^ In the Kampala registry, completeness varied by patient age and cancer site.^21^ Because of differences in the analysis approach and cancer registry type (ie, population versus regional, length of time collecting data), direct comparisons were not possible. However, we assert that the KCR is off to a strong start.

Our validation project suggests that the KCR adheres to standardized data definitions and that collected data are concordant with medical record charts, an indicator of data quality. However, a limitation of this analysis is that we only reviewed the quality of KCR contents. We did not assess how well the KCR is performing with regard to the identification of all possible cancer cases within its catchment area. The KCR is in the initial phases of collecting information from three community-based clinics that refer cancer cases to Kilimanjaro Christian Medical Center. Cancer registries will drive to community-based sites at regular intervals, abstract information on site, and then aggregate and consolidate that data with the main KCR database. Because KCR historically has focused on pathology-based case ascertainment, expansion will require a shift for inclusion of cancer cases that are only diagnosed clinically. Although there will be a transitional pilot phase, once standardized, KCR data have the potential to be more representative of all cancers diagnosed in the region. As the registry expands to collect data at additional community-based clinics and referring hospitals, validation of how well the KCR performs with regard to complete case ascertainment is an important step.

Once the quality of a cancer registry has been established, an increase in engagement with policymakers is crucial for cancer control planning and to promote future cancer registration. Registries with support from policymakers may receive additional resources and be better equipped to collect high-quality, representative data. Moreover, policymakers should incorporate cancer registry data into cancer control plan decision making. Even when registry data are collected at the hospital level, if they are of quality, they may be useful to inform local planning. In summary, the evaluation of registry quality, whether hospital or population based, is an important starting point for ensuring data integrity and should be a prerequisite for data that may be used as a basis for cancer control planning and health policy decisions.

## References

[1] Torre LA, Bray F, Siegel RL (2015). Global cancer statistics, 2012. CA Cancer J Clin.

[2] Farmer P, Frenk J, Knaul FM (2010). Expansion of cancer care and control in countries of low and middle income: A call to action. Lancet.

[3] Parkin DM, Sitas F, Chirenje M (2008). Part I: Cancer in Indigenous Africans—burden, distribution, and trends. Lancet Oncol.

[4] Parkin DM, Bray F, Ferlay J, et al: Cancer in Africa 2012. Cancer Epidemiol Biomarkers Prev 23:953-966, 2014 10.1158/1055-9965.EPI-14-0281 24700176

[5] International Agency for Research on Cancer: Cancer Incidence in Five Continents, Volume IX, 2007; http://www.iarc.fr/en/publications/pdfs-online/epi/sp160/CI5vol9.pdf

[6] Crocker-Buque T, Pollock AM (2015). Appraising the quality of sub-Saharan African cancer registration systems that contributed to GLOBOCAN 2008: A review of the literature and critical appraisal. J R Soc Med.

[7] Curado MP, Voti L, Sortino-Rachou AM (2009). Cancer registration data and quality indicators in low and middle income countries: Their interpretation and potential use for the improvement of cancer care. Cancer Causes Control.

[8] Jedy-Agba EE, Curado MP, Oga E (2012). The role of hospital-based cancer registries in low and middle income countries—The Nigerian Case Study. Cancer Epidemiol.

[9] al-Haddad BJ, Jedy-Agba E, Oga E (2015). Comparability, diagnostic validity and completeness of Nigerian cancer registries. Cancer Epidemiol.

[10] Zullig LL, Muiruri C, Abernethy A (2013). Cancer registration needs assessment at a tertiary medical centre in Kilimanjaro, Tanzania. World Health Popul.

[11] Zullig LL, Vanderburg SB, Muiruri C (2014). Sustainability of cancer registration in the Kilimanjaro Region of Tanzania—a qualitative assessment. World Health Popul.

[12] Tanzania National Bureau of Statistics: Population of Tanzania by sex, number of households, average household size and sex ratio, 2012. http://50.87.153.5/∼eastc/sensa/index.php/home/BookOneDo

[13] International Association of Cancer Registries: CanReg5. http://www.iacr.com.fr/index.php?option=com_content&view=article&id=9:canreg5&catid=68&Itemid=445

[14] National Cancer Institute, Surveillance, Epidemiology, and End Results Program: Multiple Primary and Histology Coding Rules, 2007. http://seer.cancer.gov/tools/mphrules/2007_mphrules_manual_08242012.pdf

[15] Stefan DC. Cancer care in Africa: An overview of resources. J Glob Oncol 10.1200/JGO.2015.000406 [epub ahead of print on September 23, 2015] 10.1200/JGO.2015.000406 PMC5551648 28804769

[16] Sayed S, Lukande R, Fleming KA. Providing pathology support in low-income countries. J Glob Oncol 10.1200/JGO.2015.000943 [epub ahead of print on September 23, 2015] 10.1200/JGO.2015.000943 PMC5551652 28804765

[17] Muir CS, Percy C (1991). Cancer registration: Principles and methods. Classification and coding of neoplasms. IARC Sci Publ.

[18] Bray F, Znaor A, Cueva P (2014). Planning and developing population-based cancer registration in low- and middle-income settings. IARC publication.

[19] Hofferkamp J (ed): Standards for Cancer Registries, Volume III: Standards for Completeness, Quality, Analysis, Management, Security and Confidentiality of Data. Springfield, IL, North American Association of Central Cancer Registries, 2008

[20] Shimakawa Y, Bah E, Wild CP

[21] Parkin DM, Wabinga H, Nambooze S (2001). Completeness in an African cancer registry. Cancer Causes Control.

